# Functional capacity vs side effects: treatment attributes to consider when individualising treatment for patients with rheumatoid arthritis

**DOI:** 10.1007/s10067-021-05961-8

**Published:** 2021-10-15

**Authors:** Karin Schölin Bywall, Bente Appel Esbensen, Marta Lason, Marie Heidenvall, Inger Erlandsson, Jennifer Viberg Johansson

**Affiliations:** 1 Department of Public Health and Caring Sciences, Centre for Research Ethics & Bioethics, Uppsala University, Uppsala, Sweden; 2 Copenhagen Center for Arthritis Research (COPECARE), Center for Rheumatology and Spine Diseases, Centre of Head and Orthopaedics, Rigshospitalet, Denmark; 3 Department of Clinical Medicine, Faculty of Health and Medical Sciences, University of Copenhagen, Copenhagen, Denmark; 4 Elsa Science, Stockholm, Sweden; 5 Rheumatism Association, Stockholm, Sweden; 6 Institute for Future Studies, Stockholm, Sweden

**Keywords:** Discrete choice experiment, Individualisation of treatment, Patient preferences, Rheumatoid arthritis, Shared decision-making

## Abstract

**Introduction:**

Individualisation of rheumatoid arthritis (RA) treatment needs to take account of individual patients’ preferences to increase patient-centeredness in treatment decisions*.* The aim of this study was to identify patient-relevant treatment attributes to consider when individualising treatment for patients with RA.

**Method:**

Patients with RA in Sweden were invited to rank the most important treatment attributes in an online survey (April to May 2020). Semi-structured interviews were conducted (October to November 2020) to further identify and frame potential attributes for shared decision-making. The interviews were audio-recorded, transcribed and analysed using thematic framework analysis. Patient research partners and rheumatologists supported the selection and framing of the treatment attributes across the assessment.

**Results:**

The highest ranked attributes (*N* = 184) were improved functional capacity, reduced inflammation, reduced pain and fatigue and the risk of getting a severe side effect. The framework analysis revealed two overarching themes for further exploration: treatment goals and side effects. ‘Treatment goals’ emerged from functional capacity, revealing two dimensions: physical functional capacity and psychosocial functional capacity. ‘Side effects’ revealed that mild and severe side effects were the most important to discuss in shared decision-making.

**Conclusions:**

Functional capacity (physical and psychosocial) and potential side effects (mild and severe) are important treatment attributes to consider when individualising RA treatment. Future research should assess how patients with RA weigh benefits and risks against each other, in order to increase patient-centeredness early on the treatment trajectory.

**Table:** 

Key Points *• It is essential for the individualisation of treatment to identify what attributes patients with RA are willing to trade off in order to increase patient-centeredness in precision care.* *• Individualisation of rheumatoid arthritis treatment needs to account for patients’ preferences on functional capacity and side effects.* *• Future research is needed to assess how patients with rheumatoid arthritis weigh functional capacity against side effects, in order to increase patient-centeredness in treatment decisions.*	

## Introduction

Finding the best treatment for an individual patient can be challenging, especially in conditions that can be treated with many different medicines. Rheumatoid arthritis (RA) is one such condition: an inflammatory autoimmune disease, usually treated with disease-modifying antirheumatic drugs (DMARDs), which target joint inflammation in various different ways [1]. This creates differences that may affect patients’ daily life, for instance, in administration, effectiveness and side effects. The uncertainty around treatment response in RA has resulted in a trial-and-error approach to identifying the best treatment. This can be both exhausting to patients and wasteful of healthcare resources. The major weakness of the current treatment approach is the lack of biomarkers for immediate stratification of an individual patient to the most appropriate medicine [2]. Therefore, a more individualised treatment approach called ‘precision care’ attempts to find biomarkers through ‘omics’ to match patients with therapies which they are likely to respond to, using prediction algorithms [3].

All available treatment alternatives require trade-offs between potential benefits and risks. The question remains regarding how to identify the best treatment for an individual patient. Therefore, it is essential for individualisation of treatment with DMARDs to identify what attributes patients with RA are willing to trade off, in order to increase patient-centeredness early on the treatment trajectory [4–7]. There is a large body of existing literature on patient preferences for RA treatment from a clinical standpoint [8–10]. Such studies focus mainly on endpoints relating to decreasing the number of tender and swollen joints [2]. However, individualisation of RA treatment may also consider how the treatment impacts a patient’s daily life, information that may not be included in prediction algorithms. Potentially, quantitative assessments of patient-relevant benefits and risks may be considered in precision care, to facilitate shared decision-making by aligning prediction algorithms with patients’ preferences [11–13].

Individualisation of RA treatment needs to account for individual patients’ preferences to increase patient-centeredness in treatment decisions*.* Therefore, the aim of this study was to identify patient-relevant treatment attributes to consider when individualising treatment for patients with RA.

## Material and methods

We used a step-wise approach in the identification and selection of treatment attributes: (1) attribute identification, (2) attribute ranking, (3) semi-structured interviews, and (4) attribute framing.

The attribute and level identification was influenced by the report for the Good Research Practices for Conjoint Analysis Task Force [14]. The identification began with a scoping literature review, followed by attribute filtration and validation discussions with rheumatologists and patient research partners. Nine potential attributes were ranked by patients with RA to further guide identification and selection. Semi-structured interviews were conducted to target and frame the most important attributes and levels. Refinement of the potential attributes and levels was carried out via discussions within the research team and with external rheumatologists.

### Attribute identification: step 1

A total of 373 articles were screened to identify relevant treatment characteristics (attributes) for patients with rheumatoid arthritis through a scoping literature review. Of these articles, 23 articles were eligible for inclusion in attribute identification [1, 6, 8, 9, 15–33]. Attribute selection was based on the relevance to the decision context (i.e. shared decisions in precision medicine for patients with RA) [14].

The scoping literature review revealed that that there was already a large body of existing knowledge on patient preferences from a clinical standpoint. Moreover, a large number of attributes had been assessed in previous research. Therefore, patients were asked to rank these attributes and to narrow down the number of potential attributes.

In this stage, attributes were sorted into categories: administration, treatment effects and side effects (Table 1). Administration included the potential attributes ‘route of administration’ and ‘frequency of administration’. Treatment effects encompassed ‘reduction in the number of swollen joints’, ‘improvement in functional capacity’, ‘reduced inflammation’ and ‘pain relief’. Side effects included ‘mild short-term side effects’, ‘long-term side effects’ and ‘severe side effects’. The potential attributes were discussed with rheumatologists and two patient research partners from the Swedish Rheumatism Association (MH and IE). Nine potential attributes were included in step 2.

**Table 1:** Attribute identification from the scoping literature review

Organised categories	Attribute found in review	Attributes for ranking
Administration	Route of administration Combination therapy Medication burden Frequency of administration Dose frequency	Route of administration
Treatment effects	Duration of effect Time to onset of drug effect Chance of a major symptom improvement Chance of benefit Going into remission Efficacy Radiographic progression	Reduced inflammation
	Improvement in ability to perform daily tasks Improvement in physical function	Improved in functional capacity
	Pain relief Fatigue	Reduced pain and fatigue
Side effects	Headache Nausea Vomiting Diarrhoea Injection site reaction Abnormal laboratory results	Risk of mild side effects
	Rash Oral ulcers Alopecia Weight changes Acne	Risk of side effects leading to changed appearance
	Emotional well-being Spiritual well-being Aspects of participation Independence	Risk of psychological side effects
	Pneumonitis Risk of a serious side effect Possible rare lung or liver reaction Major toxicity Risk of tuberculosis Cancer Extremely rare adverse events	Risk of severe side effects
	Risk of serious joint damage within 10 years Bone erosion	Risk of damage in the long term

### Attribute ranking: step 2

A ranking exercise was chosen as the next step to narrow down the long list of potential attributes. Patients with RA in Sweden were asked to rank nine treatment attributes in an online survey via a mobile application (www.elsa.science.se). Users of the mobile application received an invitation to participate in the ranking exercise in April 2020; the data collection lasted until the end of May 2020. Patients were eligible for the ranking exercise if 18–80 years of age, an established RA diagnosis and able to understand the questions without aid. In total, 262 potential respondents started the survey, of whom *n* = 184 were included in the final analysis. Responders were excluded if not providing informed consent before accessing the survey. The survey was approved by the regional ethics review board in Uppsala, Sweden (Reg no. 2020/00556). Data collection and recording, storage and dissemination were governed by the General Data Protection Regulation (GDPR) and Uppsala University’s data protection and security policies.

The attributes in the ranking exercise were presented to each respondent in a random order. Respondents ranked all of the attributes, with the most important attribute (i.e. highest ranked) given 9 points and the rest of the attributes given a decreasing number, down to 1 point. We used the *χ*^2^ test to explore if there was any significant differences in patient preferences related to disease duration, age, education or health literacy [34]. Disease duration was dichotomised as under 10 years of RA and over 10 years of RA. The cut-off for age was 45 years. Patient research partners assisted in the development of the semi-structured interview guide, to explore the results of the ranking exercise with the aim of identifying patient-relevant attributes and levels to be assessed in a quantitative patient preference assessment, such as a discrete choice experiment [10].

Health literacy was calculated for each respondent [35]. Individuals responding with strongly disagree or disagree to one of the items were categorised as having inadequate HL. Individuals responding with neither agree nor disagree to one of the items were categorised as having problematic HL. Finally, individuals responding agree or strongly agree to all the items were categorised as having sufficient HL.

### Semi-structured interviews: step 3

Semi-structured interviews were chosen as the next step to get a deeper understanding of the results from the ranking exercise. Respondents in the ranking exercise were asked to participate in semi-structured interviews (October to November 2020) via a mobile application (www.elsa.science.se). Inclusion criteria’s for the interviews were 18 years of age, an established RA diagnosis less than 5 years ago and able to understand and answer the interview questions without aid. Potential respondents were excluded if they had RA for more than 5 years. The interviews were conducted by KSB, audio-recorded and transcribed. Each interview was scheduled for 1 h. The analysis was performed by the authors KSB, MH, IE, BAE and JVJ. The thematic framework analysis method was chosen for the analysis, as it has the potential to support multiple research teams where not all research team members have experience of qualitative data analysis. This method provides steps to follow and produces structured outputs of summarised data [36]. The framework analysis was initiated by coding meaning units of the transcribed interviews. Each code described some contents of the text. The analytical framework was developed alongside performance and analysis of the interviews. Data saturation was discussed among the coders and concluded after 10 interviews. Codes were grouped into categories under the overarching themes: administration, treatment effects and side effects (Table 1). The framework was applied to and refined for each transcript in a structured manner. Each category was then summarised based on quotes for the codes. These category summaries were considered in the development of potential attributes to consider in shared decision-making. The framework analysis was also discussed with two rheumatologists, to reflect clinical practices.

### Attribute and level framing: step 4

Validation interviews were conducted to test the framing of the attributes and to assign levels to the attributes. An invitation was sent to the respondents by email, with a link to the survey. All respondents provided informed consent. Interviews were conducted digitally with three patients with RA. Some of the attribute framing was adjusted after this. The attribute refinement also included discussions with two rheumatologists and the research team.

## Results

### Attribute identification: step 1

The attribute identification resulted in nine potential attributes relevant for the quantitative patient preference assessment. Three attributes related to treatment effect: ‘the treatment’s ability to increase my functional capacity to have an active lifestyle’,’the treatment’s ability to reduce inflammation in my joints’ and ‘the treatment’s ability to decrease my pain and fatigue’. Five of the attributes related to potential side effects: ‘to avoid mild short-term side effects such as nausea and headache’; ‘to avoid damage in the long term such as arteriosclerosis or osteoporosis’; ‘to avoid severe side effects such as infections leading to me being hospitalised’; ‘to avoid side effects that can affect my mental health, such as mood changes or sleep disturbance’; and ‘to avoid side effects that can alter the way I look, such as skin rash or weight change’. Route of administration and frequency of administration were merged into one, to reduce the number of attributes.

### Attribute ranking: step 2

Most of the respondents were female (93.5%), with a disease duration over 10 years (66.3%). The average age span was between 45 and 64 years (56%). Responders were generally highly educated, with 59.3% having studied at university (Table 2).

**Table 2:** Demographic characteristics

Disease duration		*n* = 184	%
	0–3 months	2	1.1
	3–6 months	3	1.6
	6–12 months	7	3.8
	1–2 years	14	7.6
	2–3 years	18	9.8
	3–5 years	6	3.3
	5–10 years	12	6.5
	10 + years	122	66.3
Age (years)	18–24	10	5.4
	25–34	14	7.6
	35–44	22	12
	45–54	50	27.2
	55–64	53	28.8
	65 +	35	19
Gender			
	Female	172	93.5
	Male	12	6.5
Education			
	Elementary school 9 years	12	6.5
	2-year high school	21	11.4
	3–4-year high school	31	16.8
	College	11	6
	University less than 3 years	36	19.6
	University more than 3 years	73	39.7
Health literacy			
	Insufficient	13	7
	Problematic	74	40
	Sufficient	97	53

**Fig. 1:**
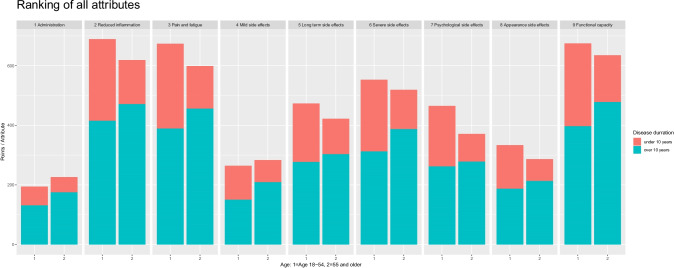
Attribute ranking stratified on disease duration and age

The attribute rankings are summarised as presented in Fig. 1. The most important attribute was ‘improved functional capacity’, which was given 1,310 points, with the second most important being ‘reduced inflammation’, given 1,308 points. The third was ‘reduced pain and fatigue’ (1,273 points), followed by ‘risk of severe side effects’ (1,072 points), long-term side effects (895 points), risk of psychological side effects (836 points), risk of side effects affecting appearance (619 points), risk of mild short-term side effects (547 points) and route of administration (420 points). The *χ*^2^ test showed that there was no statistically significant differences in patients’ rankings in relation to disease duration, age, education or health literacy. Figure 1 illustrates the attribute rankings and that there were no statistically significant differences when strafing the data on disease duration of RA and age. The bar charts present two dichotomised age categories, 1 = 18–54 and 2 = 55 and older for each attribute. Each bar chart presents dichotomised disease duration in two colours, bottom = over 10 years of RA and top = under 10 years of RA.

### Semi-structured interviews: step 3

In total, 10 interviews were conducted (*n* = 8 female). Respondents represented several geographic locations in Sweden. The first interview was considered a pilot interview, with the purpose to test the interview guide. The respondents represented people from all age ranges in the ranking exercise. All of the respondents had been diagnosed with RA within the last 5 years and were chosen to represent the views of relatively recently diagnosed patients.

Two overarching themes emerged from the framework analysis: treatment goals and side effects (Table 3). ‘Treatment goals’ emerged from the main categories ‘physical treatment goals’ and ‘psychosocial treatment goals’. Physical treatment goals emerged from the codes ‘inflammation’, ‘swollen joints’, ‘pain’ and ‘physical fatigue’. One of the respondents said: ‘The most important thing for me is to function, yeah to be able to work, and do the things I am used to and such’. Psychosocial treatment goals emerged from the codes ‘social ability’, ‘mental ability’, ‘pain’ and ‘psychosocial fatigue’. One respondent said: ‘I avoided doing things, if you feel bad you don’t want to meet people’. The attributes ‘improved physical functional capacity’ and ‘improved psychosocial functional capacity’ emerged from patients’ individual treatment goals. These attributes included elements of several attributes from the ranking exercise: reduced inflammation, improved functional capacity, reduced pain and fatigue.

**Table 3:** Framework analysis of semi-structured interviews to identify attributes

	Codes	Sub-categories	Categories	Quotes (selection)
Overarching theme	Treatment goals: physical			
	Inflammation	Disease impact on physical functions	Physical treatment goals	IP6: ‘The most important thing for me is to function, yeah to be able to work, and do the things I am used to and such’
	Swollen joints			
	Pain and fatigue physical			
Summary	Physical functional capacity is important to keep up with daily life activities			
Attribute	**Improved physical functional capacity** (my ability to perform daily tasks and activities, such as work, studies, household, family and spare time)			
Overarching theme	Treatment goals: psychosocial			
	Social ability	Psychosocial function	Psychosocial treatment goals	IP6: ‘So that is a limitation and then, I avoided doing things, if you feel bad you don’t want to meet people’
	Mental ability			
	Pain and fatigue psychosocial			
Summary	Psychosocial functional capacity can impact well-being			
Attribute	**Improved psychosocial functional capacity** (how I feel and my ability to have a functional social life)			
Overarching theme	Side effects: severe			
	Unsafe	Severe side effects	Acute severe side effects	IP6: ‘You don’t want to get severely ill in something else’
	Immediate	Acute side effects		
	Future	Long-term side effects	Permanent long-term side effects	
	Damage	Permanent side effects		
Summary	Patients do not like severe side effects, such as pneumonia			
Attribute	**Severe side effects** (severe infection or allergic reaction)			
Overarching theme	Side effects: mild			
	Nausea	Transient mild side effects	Transient mild side effects	IP6: ‘One of the medicines I took gave me nausea the whole day after’
	Headache			
	Mood changes	Psychological		
	Weight changes	Appearance		
	Hair loss			
Summary	Mild side effects may affect a patient’s daily life			
Attribute	**Mild side effects** (nausea or headache)			

The theme ‘side effects’ emerged from the categories ‘transient mild side effects’, ‘permanent long-term side effects’ and ‘acute severe side effects’. Each of the categories was framed as a potential attribute in the preference assessment. Examples of mild side effects were nausea or headache (i.e. excluding the sub-categories related to psychological side effects and appearance). One respondent said: ‘One of the medicines I took gave me nausea the whole day after’. For the attribute ‘likelihood of a severe side effect’, the examples used were severe infections or allergic reactions.

### Attribute framing: step 4

Some of the attributes were adjusted to improve the framing. Levels were assigned to the attributes to support the setting of individual treatment goals as part of shared decision-making. The attribute refinement process also included discussions with two rheumatologists and the research team.

Physical functional capacity was framed as ‘my ability to perform daily tasks and activities, such as work, studies, household, family and spare time’. Psychosocial functional capacity was framed as ‘how I feel and my ability to have a functional social life’. Both attributes were assigned the levels 25, 50, 75 and 100 (full function).

The attribute ‘mild side effects’ was framed as the frequency of getting nausea or headache. Three levels for mild side effects were identified and adjusted after discussion: often (weekly), sometimes (monthly) and rarely (quarterly).

Severe side effects were framed as the likelihood of getting a severe side effect, such as a severe infection or allergic reaction. Three levels were assigned after attribute refinement: common (1 in 10 can get the side effect), uncommon (1 in 100 can get the side effect) and rare (1 in 1,000 can get the side effect) to reflect the information given on the package leaflets for existing DMARDs.

## Discussion

The aim of this study was to identify patient-relevant treatment attributes to consider when individualising treatment for patients with RA. We outlined an explorative approach to identify patient-relevant treatment attributes by using both quantitative ranking and following up the results in semi-structured interviews. The reason for taking this approach was the large body of existing knowledge from a clinical standpoint. The attribute ranking generated a first insight into what attributes were most important to patients. We decided to follow these results in further attribute exploration through semi-structured interviews with patients for further exploration. We identified four treatment attributes, with 3–4 levels each, as being relevant for patients with RA in precision care. The attributes were related to functional capacity (physical and psychosocial) and potential side effects.

Results from the ranking exercise were in line with previous research in the same field regarding preferences among patients with RA [1, 6, 8, 9, 15–33]. However, we decided to exclude attributes related to treatment administration, as the route of administration is seldom the most important attribute in these studies and was ranked lowest in this study. Administration methods may differ between different treatment alternatives, and there may sometimes be a need for tight control in order to find a suitable treatment. The relevance of such practical issues should not be underestimated, as treatment must fit into a patient’s daily life. Therefore, there may be a need for clinicians to broach such matters in discussions with patients.

One of the most important treatment attributes seen in the larger literature body was treatment effect [37]. Treatment effect is commonly assessed in terms of ‘improvements’, ‘chance of efficacy’ or ‘duration of effects’ [9]. Treatment effect reflects the attribute ‘reduced disease activity’ selected in the ranking exercise. Effectiveness was an attribute among treatment goals, in terms of physical and psychosocial functional capacity. Physical functional capacity has previously been addressed in research as the patient’s wish for ‘a normal life’ through symptom relief [38]. Physical functional capacity has previously been assessed as ‘improvement in ability to perform daily tasks and activities’ [25], and ‘psychosocial functional capacity’ has been assessed as well-being, both physical, mental, emotional and spiritual [28, 30]. Similar attributes for mild side effects have previously been assessed as ‘risk of immediate mild treatment reaction’ [26], and severe side effects are commonly measured in frequencies of ‘potential risks’ [29]. The results of this study suggested that attributes serving to support patients in treatment individualisation need to reflect patients’ own treatment goals and preferences rather than taking a clinical standpoint. Therefore, shared decision-making in RA treatment should consider attributes that influence a patient’s daily life and quality of life, to support patients in individualisation of care.

A strength of this study was that we were able to include a large number of respondents in the ranking exercise. The process was also supported by rheumatologists and patient research partners. However, a limitation of this study may have been that recruitment via a mobile application could exclude certain groups of patients who do not use such applications. Therefore, the results of this study may not be generalisable to the general RA population in Sweden.

Recommendations for precision care should include data from the patient perspective, to support patients in shared decision-making. Methods for assessing and using patient preferences in order to strengthen patients in shared decision-making in precision care are lacking. Quantitative assessments of patient-relevant benefits and risks may support patients in shared decision-making, so treatment decisions can be aligned with their preferences. Future research should assess how patients with RA weigh benefits and risks against each other in order to increase patient-centeredness early on the treatment trajectory.

## Conclusions

This study contributes to a deeper understanding of what is important to patients in treatment individualisation. Treatment attributes important to patients were related to improving functional capacity and acceptable side effects. Quantitative assessments of patient preferences should consider treatment effects on patients’ own treatment goals and the impact that treatment has on daily life activities. Future research is needed to support the use of patient preferences in order to strengthen patients in individualisation of care.

## Data Availability

Data from the current study is available from the corresponding author on reasonable request.
